# Patient Portal Use, Perceptions of Electronic Health Record Value, and Self-Rated Primary Care Quality Among Older Adults: Cross-sectional Survey

**DOI:** 10.2196/22549

**Published:** 2021-05-10

**Authors:** Dori A Cross, Zachary Levin, Minakshi Raj

**Affiliations:** 1 Division of Health Policy and Management University of Minnesota School of Public Health Minneapolis, MN United States; 2 Department of Kinesiology and Community Health University of Illinois at Urbana-Champaign Urbana-Champaign, IL United States

**Keywords:** patient portals, electronic health records, primary care, older adults, patient satisfaction

## Abstract

**Background:**

Older adults are increasingly accessing information and communicating using patient-facing portals available through their providers’ electronic health record (EHR). Most theories of technology acceptance and use suggest that patients’ overall satisfaction with care should be independent of their chosen level of portal engagement. However, achieving expected benefits of portal use depends on demonstrated support from providers to meet these expectations. This is especially true among older adults, who may require more guidance. However, little is known about whether misalignment of expectations around technology-facilitated care is associated with lower perceptions of care quality.

**Objective:**

The aims of this study were to analyze whether older adults’ assessment of primary care quality differs across levels of patient portal engagement and whether perceptions of how well their provider uses the EHR to support care moderates this relationship.

**Methods:**

We conducted a cross-sectional survey analysis of 158 older adults over the age of 65 (average age 71.4 years) across Michigan using a 13-measure composite of self-assessed health care quality. Portal use was categorized as none, moderate (use of 1-3 functionalities), or extensive (use of 4-7 functionalities). EHR value perception was measured by asking respondents how they felt their doctor’s EHR use improved the patient–provider relationship.

**Results:**

Moderate portal users, compared to those who were extensive users, had lower estimated care quality (–0.214 on 4-point scale; *P*=.03). Differences between extensive portal users and nonportal users were not significant. Quality perception was only particularly low among moderate portal users with low EHR value perception; those with high EHR value perception rated quality similarly to other portal user groups.

**Conclusions:**

Older adults who are moderate portal users are the least satisfied with their care, and the most sensitive to perceptions of how well their provider uses the EHR to support the relationship. Encouraging portal use without compromising perceptions of quality requires thinking beyond patient-focused education. Achieving value from use of patient-facing technologies with older adults is contingent upon matched organizational investments that support technology-enabled care delivery. Providers and staff need policies and practices that demonstrate technology adeptness. Older adults may need more tailored signaling and accommodation for technology to be maximally impactful.

## Introduction

Patients’ increased access to, and engagement with, their digitized health information has featured prominently in recent federal law and quality-based payment programs designed to accelerate value-generating use of health information technology [1,2]. The Medicare and Medicaid Electronic Health Record (HER) Incentive Programs have helped to advance use of patient portal technologies as a mechanism for information access and electronic patient–provider communication. Portals are intended to increase patient engagement in managing their own health and health care needs, and improve accessibility and efficiency of receiving certain clinical advice, reminders, and services [3-5]. However, evidence linking portal use to improved measures of quality or value is mixed [6-9]. This uncertainty regarding the value of portal use is especially true for older adults, a population for which there has been substantial attention concerning lag in adoption [10] and less understanding of variation in patterns of portal use and perceptions of information technology–enabled care [11-14]. More recent evidence suggests some narrowing of the digital divide based on age [12,15] and significant potential for enhanced communication and self-management engaging older adults through portal technologies [13,16,17]. However, the “right” level of portal use to encourage among older adults is difficult to pinpoint and context dependent.

**Figure 1:**
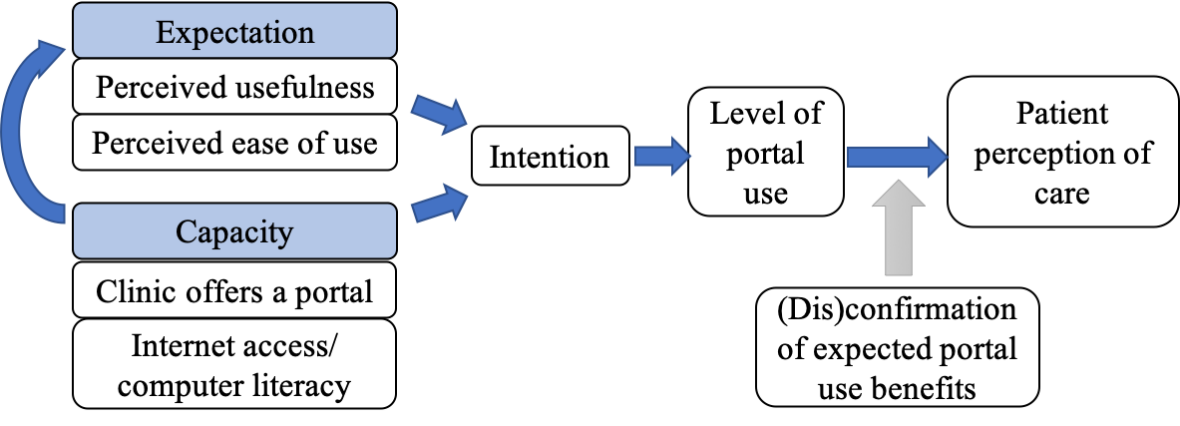
Integrated conceptual model of portal use and perception of care quality modified by expectation confirmation.

Patients, provided that they are offered portal access in the first place [15], make usage choices based on technology expectations (eg, perceived usefulness and ease of use) and enabling factors, such as the level of support, encouragement, and education received from providers on how to engage with the portal [18-20]. Together, enabling factors and expectations shape intention and actual portal use, as articulated by the technology acceptance model and the more updated Unified Theory of Acceptance and Use of Technology [21,22] (see Figure 1). However, the linkage between level of portal use and patients’ overall satisfaction with care is ill defined.

Empirically, there appears to be little association between how much a portal is used (ie, a dose response) and objective measures of quality (eg, hemoglobin A_1c_ control, readmissions) [23,24]. A key theoretical consideration is that portals are a shared tool between patients and providers. A patient’s perception of benefit from using the portal is reevaluated with every experience the user has in using the portal to facilitate communication and needed services with a health care provider. Whether a patient is happy with care at their chosen level of portal engagement is therefore dependent on whether the experience matches their expectation [25,26]. Extending the technology acceptance model with expectation–confirmation theory (see Figure 1) reflects this more interactive understanding [27]. (Dis)confirmation of patient expectations around the use of portals, shaped by how the provider organization is engaging from their end, may modify the relationship between chosen levels of engagement and patients’ self-assessment of care quality.

Among older adults, the extent to which their level of portal engagement is matched by perceived reciprocal investment by their provider is a heightened contextual factor that may influence how older adults assess their quality of care. Older adults’ expectations for how their primary care providers can structure and deliver health care in a more technology-advanced way continue to evolve, and their perceptions of this alignment in investment is heightened given the additional guidance and support that older adults more often need in order to feel comfortable in engaging with patient-facing technologies [28,29]. Observations about how the provider uses the EHR in-clinic—perceived adeptness of use, continuity or disruption of attention and communication, and whether providers voice frustration with use—meaningfully shape whether patients perceive that their provider’s use of technology adds value to, or detracts from, the value of their care [30-32]. To the extent that older adults’ value assessments around portal engagement may be tied to perceptions of how effectively their providers use the EHR, we must consider these as possibly interdependent factors that shape overall assessment of care quality [29,31,33].

This study used a novel dataset of older adults surveyed about their health needs, provider relationships, and perceptions of health system experiences. We characterized the nature of portal engagement among a sample of older adults and then addressed the following 2 specific research questions: (1) Are older adults’ assessments of primary care quality independent of the level of personal engagement with patient portal technologies? (2) To what extent do patient perceptions of how their provider uses technology modify observed relationships between extent of patient portal use and assessment of care quality? Ultimately, this paper seeks to provide actionable insights that help providers leverage health information technology investment for improved experiences of primary care for a large and growing older adult population.

## Methods

### Setting and Data Source

A statewide survey regarding older adults’ perceptions of health care needs and navigation of primary care services was developed and administered between March 2019 and June 2019 across Michigan, which has population demographics that closely represent nationwide aging trends [34]. The survey instrument was informed by 4 focus groups with a total of 18 older adult participants, and drew upon elements of previously validated surveys, including the clinician/group survey of the Consumer Assessment of Healthcare Providers and Systems (CAHPS) and the National Poll on Healthy Aging [35,36]. “Think aloud” cognitive testing was then conducted with an additional 10 participants for survey refinement to ensure functionality, appropriate wording, and comprehension [37,38]. We used this adaptive measure development approach because many validated measures of primary care quality are general to the adult population; this broader survey effort sought to define quality from the specific perspective of aging. [39] This survey study was reviewed and determined to be exempt from full human subjects review by the University of Michigan Institutional Review Board. The full survey instrument is available upon request.

Participants were recruited statewide using an online health research platform managed by a large academic medical center with a pool of roughly 50,000 prospective participants [40]. Respondents needed to be over 65 years of age, comfortable with written English, have a person whom they consider their “regular doctor”, and have passed a brief cognitive test. All respondents must have received health care in the United States at least once in the past year but not necessarily with their regular doctor. Because we used a convenience sampling approach, we collected a number of demographic and health status indicators to help characterize our final sample and appropriately limit the generalizability of our findings [14,16]. Participants received a brief overview of the study and the URL for accessing the survey via Qualtrics where they also provided written informed consent. Respondents were asked to reflect specifically on health care experiences with the person they consider their primary care provider. Survey sections containing the questions of interest for our analyses were distanced from each other within the instrument, and question order within each section was randomized across participants [41]. Participants received US $10 for participation.

### Measures

#### Outcomes

Our primary outcome was a composite measure of participants’ overall self-rated health care quality. The survey contained 13 questions that assessed dimensions of quality. Four of these questions were adopted from the CAHPS patient survey [36]; 9 additional questions assessing care coordination, patient-centered care experience, and age-sensitive care delivery were added based on focus group findings to reflect the particular experiences of older adults (Multimedia Appendix 1). All questions were assessed on the same 1-4 scale (“not true,” “somewhat true,” “fairly true,” or “very true”) as preferred by respondents during cognitive testing and reverse coded as necessary. We averaged the values provided across all 13 questions to assign each respondent a summary value between 1 and 4. The Cronbach α reliability coefficient was .94, and exploratory factor analysis supported use of a single factor, with all questions loading on to this factor at or above 0.68 (Multimedia Appendix 2).

#### Technology Perception and Use

We used 2 health information technology–related survey questions. One measure captured how respondents use health information technology via available patient portal features. We asked about usage of 7 features across 3 domains: information availability (viewing laboratory/test results, reviewing physician’s advice, finding medications), communication (messaging with provider, answering previsit questions), and convenience (refilling medications, paying bills online). These questions were adapted from the National Poll on Healthy Aging [35]. We then created 3 categories of portal use: no portal use, moderate portal use (1-3 features used), and extensive portal use (4-7 features used).

The second question focused on perception of provider’s use of EHRs. We asked respondents, on a 1-4 scale, if they felt their doctor’s EHR use improved their patient–provider relationship. We focused on improved relationship as a measure of EHR use given our interest in the value of EHR for communication and patient engagement. We created a binary indicator for EHR value perception; participants who reported a 1 or 2 (“not true” or “somewhat true”) were labeled as having a low EHR value perception and those who reported a 3 or 4 (“fairly true” or “very true”) were labeled as having a high EHR value perception.

#### Covariates

The following self-reported demographic and clinical profile characteristics were available from survey data: age, race, sex, highest education completed, and an indicator of financial status (how often respondent has money left over at the end of the month). Clinical profile measures included the patient’s self-reported health status (poor or fair, good, and very good or excellent), an indicator of polypharmacy (ie, whether a participant self-reports taking 4 or more pills) [42], number of primary care and number of total health care encounters in the past 6 months, and whether the participant has a caregiver. We also asked respondents the duration of their relationship with their doctor (<5 years, 5-10 year, 10+ years).

#### Check for Common Method Bias

Because our main variables of interest (ie, information technology perception/use questions) and our composite quality outcome measure derived from the same survey instrument, we tested for common method bias prior to conducting any analyses [41]. Factor analysis on the combination of all included measures confirmed that there was no single latent factor accounting for most of the covariance among survey questions.

### Analytic Methods

We first calculated summary statistics for all available demographic and clinical profile characteristics of our survey respondents, and, where possible, considered comparative national statistics to understand how well our sample compared to the demographic profile of older adults across the United States. We also calculated summary statistics for our outcome measure: the 13-item composite of self-reported health care quality.

For our information technology–specific questions, we calculated the percent of respondents using each of the 7 available portal features. To understand the distinguishing characteristics of respondents at each level of portal use (eg, nonportal users, moderate portal users, extensive portal users), we assessed bivariate relationships between portal use and all available demographic and health status measures (listed above as covariates). We also tested the association between our portal use categories and the EHR value perception measure (ie, how respondents perceive value in their provider’s use of the EHR). Because health status and health care needs may influence the specific portal features considered useful to an individual, we also ran a supplemental analysis to look at whether the types of portal use (ie, which features) varied based on self-rated health status and/or extent of medication use.

We next examined the bivariate relationships between all available respondent characteristics and the composite quality measure to assess which characteristics are relevant to include in our full models. We estimated full multivariate ordinary least squares regression models with robust SEs. Examining the distribution of errors postestimation supported this model choice. We first estimated the independent adjusted effects of the extent of respondent portal use and the EHR value perception on the composite quality outcome measure. We then reran these models and included an interaction between these variables to assess whether EHR value perception moderates the association between respondents’ personal portal use and self-reported health care quality.

## Results

### Summary of Data

Our survey yielded 167 responses from adults over the age of 65, with 158 surveys sufficiently complete for full analysis. The average age of respondents in our sample was 71.4 years. Respondents were 66.5% (105/158) female, highly educated (45.2% [71/157] reporting higher than a bachelor’s degree), and in good health (57.6% [91/158] reporting very good or excellent health status; Table 1). These characteristics, in addition to a younger-skewed age distribution and underrepresentation of rural respondents, differentiate our sample relative to national corresponding statistics. About one-third of participants (46/153, 30.1%) had a formal or informal caregiver who supported their health and health care needs, and 17.9% (28/158) expressed concern with technology use. Individuals varied in clinical complexity (54/157 [34.3%] taking 4 or more pills daily) and financial security (53/158 [33.5%] reporting money rarely or sometimes left over at the end of the month). Over half (88/158, 55.7%) of the respondents had been seeing their doctor for at least 5 years.

Our 13-item composite outcome measure of perceived health care quality averaged 3.46 out of 4 across our sample (SD 0.46). Of the 158 respondents, 30 (19.0%) reported uniformly high quality on all 13 questions.

**Figure 2:**
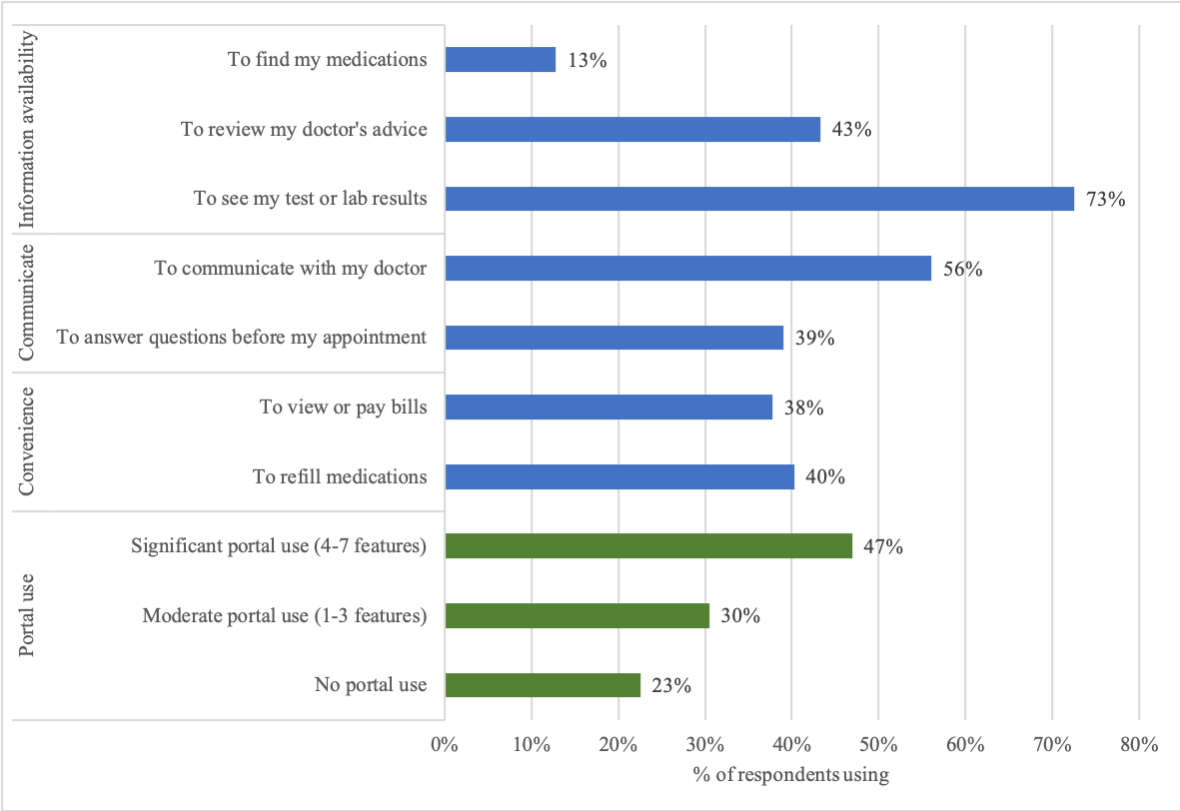
Use of available portal functions among respondents.

Portal use among this sample was relatively high. Portal use was found to be positively associated with the number of doctors a patient had seen in a 6-month time frame and negatively associated with age, technology concerns, and financial stability (Multimedia Appendix 3). Most health status characteristics (eg, self-reported health status, polypharmacy, having a caregiver) were not associated with extent of portal use. Of all respondents (N=164 with portal use data), 127 (77.4%) used at least 1 of 7 available functionalities (Figure 2). The most commonly used features were viewing test/laboratory results (72.6%, 119/164) and communicating with the doctor (56.1%, 92/164). The average respondent used 3 different functions; 22.6% (37/164) of the sample reported no portal use, 30.4% (50/164) used 1-3 features (moderate), and 47.0% (77/164) of the sample used 4 or more features (extensive). We found that individuals taking more medications were more likely to use the portal to find their medication list but less likely to use the portal for answering previsit questions. We observed no significant differences by self-reported health status (Multimedia Appendix 4).

**Figure 3:**
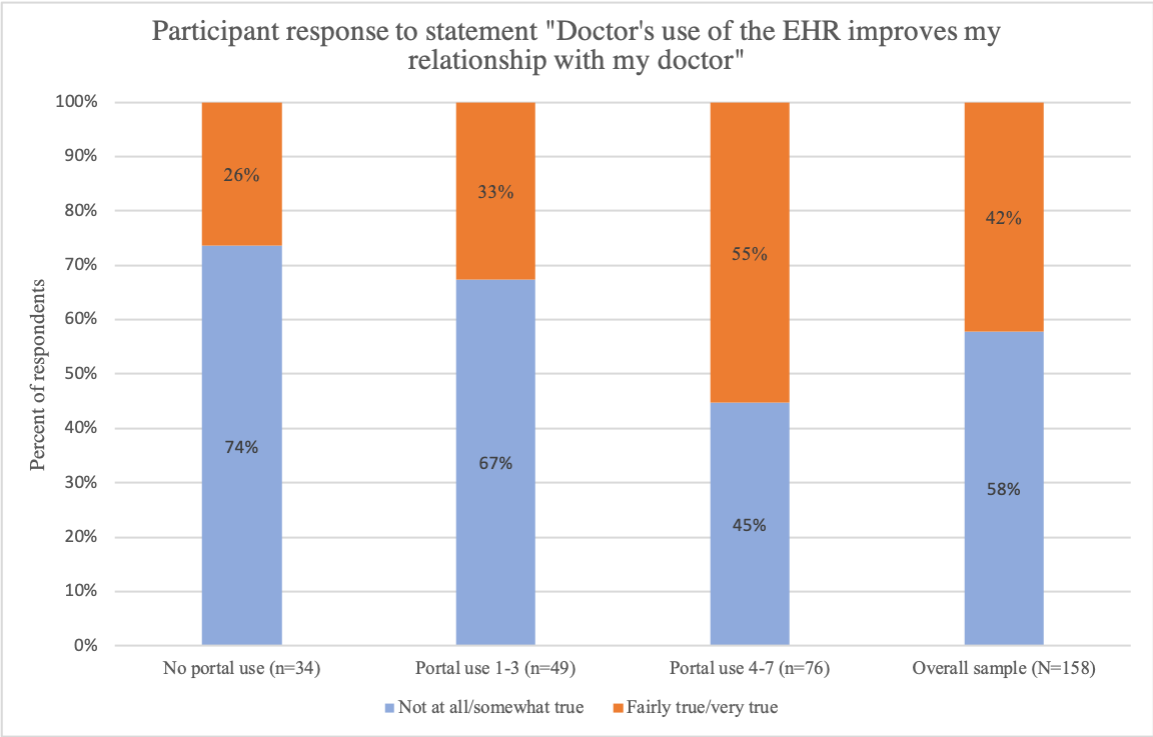
Comparison of EHR value perception by portal use category. EHR: electronic health record.

Overall, 67 respondents (41.5%) reported that the EHR improved their relationship with their physician (high EHR value perception; Figure 3). This percentage was greatest among extensive portal users (42/76, 55%). Respondents who were nonusers and moderate portal users were significantly less likely to have high EHR value perception (nonusers: 9/34, 26%; moderate users: 16/49, 33%; chi-square *P* value=.01).

**Table 1:** Respondents’ sample characteristics.

Characteristic			Survey sample (N=158), n (%)	Corresponding national statistics (%)
**Demographics**				
	**Age (years)^a^**			
		65-69	83 (52.5%)	32.6%
		70-74	35 (22.2%)	25.6%
		75-79	24 (15.2%)	17.7%
		80+	16 (10.1%)	24.2%
	Female^a^		105 (66.5%)	55.5%
	Nonmetropolitan county^b^		9 (5.7%)	14.1%
	**Highest education attained^c^**			
		< Bachelor's degree	41 (26.1%)	70.8%
		Bachelor's degree	45 (28.7%)	16.5%
		> Bachelor's degree	71 (45.2%)	12.7%
	Has money left over at the end of the month (always/often)^d^		105 (66.5%)	66.7%
	Concerns with technology use		28 (17.9%)	—^e^
**Health status**				
	**Self-reported health status^f^**			
		Fair/poor	29 (18.4%)	23.0%
		Good	38 (24.1%)	33.1%
		Very good/excellent	91 (57.6%)	43.9%
	Percent of patients taking 4+ pills daily^g^ (n=157)		54 (34.3%)	54%
	Has a caregiver (n=153)		46 (30.1%)	—
	**Patient–provider relationship**			
		Seeing personal doctor for 5+ years	88 (55.7%)	—

^a^National statistics from the US Census Bureau, Population Division (2019) [43].

^b^National statistics from the US Department of Agriculture Economic Research Service (2019) [44].

^c^National statistics from the US Census Bureau (2018) [45].

^d^National statistics from the National Council on Aging (2015) [46].

^e^Comparable national statistics could not be found.

^f^National statistics from the Centers for Disease Control and Prevention (2018) [47].

^g^National statistics from the Kaiser Family Foundation (2019) [48].

#### Regression Results

Bivariate analyses that tested association of each patient-level covariate and the composite quality outcome measure revealed only 3 significant associations: respondents’ length of relationship with their provider, self-reported health, and whether the respondent had money leftover at the end of the month. The multivariate models presented include only these controls. Models with all available covariates included had consistent findings; because of our sample size, we present the more parsimonious model.

Column 1 of Table 2 presents our noninteracted multivariate model. Relative to extensive portal users, moderate portal use was significantly associated with lower self-rated quality (–0.214 on 4-point composite scale; *P*=.03). Adjusted differences between extensive portal users and nonportal users were not significant. Estimates of self-reported quality were also significantly higher for individuals with high EHR value perception relative to low perception (0.288; *P*<.001). Among control variables, higher quality was associated with individuals having a longer (10+ years) relationship duration with their provider and with being in very good or excellent health. Effects of financial stability were not significant.

**Figure 4:**
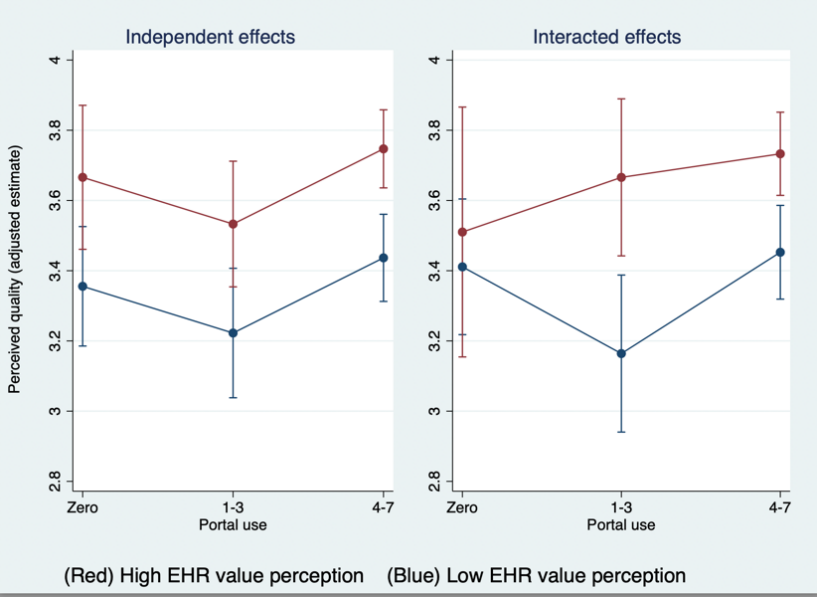
Adjusted estimates of self-rated quality by EHR value perception and portal use. EHR: electronic health record.

When we interacted EHR value perception with respondent portal use (column 2 of Table 2), we still observed high EHR value perception to be positively and significantly associated with self-rated quality, and that there were significant differences in quality only between extensive and moderate portal users (not between extensive and nonportal users). The interaction terms were not statistically significant, suggesting that these significant differences in self-rated quality by portal use category are more salient among those with low EHR value perception. Figure 4 visually demonstrates the effect of EHR value perception on the relationship between portal use and self-rated quality. In the noninteracted model (ie “independent effects”), the effect of EHR value perception remained consistent across all levels of portal use. The interacted model demonstrates differential effects where moderate portal users appear particularly sensitive to EHR value perception. Among this group, those with low EHR value perception had especially low predicted estimates of perceived quality, while those with high EHR value perception reported a more similar self-rated quality to those who were extensive portal users.

**Table 2:** Multivariate ordinary least squares regression results: adjusted estimated effects on overall patient self-rated quality (N=158).^a^

Estimated effects on self-rated quality		Model without interaction, coefficient (SE)	*P* value	Model with interaction, coefficient (SE)	*P* value
**Portal use category (reference: extensive portal use)**					
	No portal use	–0.081 (0.104)	.434	–0.041 (0.121)	.73
	Moderate portal use	–0.214 (0.095)	.027	–0.288 (0.131)	.03
High EHR^b^ value perception (reference: low EHR value perception)		0.288 (0.074)	<.001	0.257 (0.086)	.004
**Portal use x EHR value perception (reference: high EHR value perception x extensive portal use)**					
	High EHR value perception x no portal use	N/A^c^	N/A	–0.181 (0.228)	.44
	High EHR value perception x moderate portal use	N/A	N/A	0.221 (0.181)	.23
**Length of relationship with primary care physician (reference: <5 years)**					
	5-10 years	0.176 (0.097)	.071	0.173 (0.097)	.08
	10+ years	0.350 (0.086)	<.001	0.339 (0.086)	<.001
**Self-reported health (reference: very good/excellent)**					
	Poor/fair	–0.246 (0.119)	.041	–0.221 (0.123)	.08
	Good	–0.216 (0.092)	.034	–0.196 (0.092)	.06
Money left over at the end of the month always/often (reference: rarely/sometimes)		0.080 (0.099)	.422	0.091 (0.099)	.36
Constant		3.19 (0.163)	<.001	3.40 (0.151)	<.001
*R* ^2^		0.274	N/A	0.288	N/A

^a^Outcome (self-rated quality) on a 4-point scale.

^b^EHR: electronic health record.

^c^N/A: not applicable.

## Discussion

### Principal Findings

Using novel statewide survey data of older adults’ health care needs and preferences, we found that moderate portal users, those who use just a few available features, had the lowest self-rated quality, lower than both extensive portal users and nonportal users. Among this group, it was particularly those individuals also with low EHR value perception who had the lowest estimates of self-rated quality. Those with high EHR value perception had estimated quality levels similar to those of extensive portal users. These analyses preliminarily support an expectancy confirmation theory extension to patient technology acceptance. Our findings are a first step toward understanding how a mismatch between older adults’ chosen level of engagement in technology-enabled care and perceptions of how well their provider uses technology to deliver services may drive variation in how older adults assess overall quality of their primary care.

Our results reveal an important emerging dual consideration with older adults. The number of older adults who are interested in and capable of participating in technology-supported health care is growing especially as it enables them to age in place [49,50]. Even those with some trepidation are starting to engage with these tools, and thoughtful support of this experience is increasingly a key component of how they perceive their care experience. These considerations now exist alongside longstanding concerns for a significant number of older adults who still have very salient individual and structural barriers to engagement (to the point that they are not even meaningfully captured via a primarily online survey like what has been presented in this study). Both issues merit attention and investments in improvement.

Fostering higher and more equitable rates of portal engagement with older adults requires offering portal access more equitably [15] and coaching patients (and their family members) through the features while being more explicit about shared expectations and the “rules of engagement” regarding portal use [13,20]. Encouraging patient portal use without compromising perceptions of quality, however, also requires that clinics demonstrate that they are competent, proactive users of technology. This means, first, that providers and staff must convey their own commitment to using the portal as a communication tool, including more robust provider or staff training and explicit policies around responsiveness and follow-up [20,29]. Enabling and sustaining this commitment requires health care organizations to support providers in these efforts, for example, by building time for portal encounters into scheduling and productivity considerations, and using EHR features such as shared task queues to facilitate delegation of communications that do not require physician response [51].

Our results also underscore the importance of opinions that patients form regarding providers’ adeptness at using the EHR during in-person care. These opinions—in particular for those patients who are engaging with the portal but are not quite super users—are strongly associated with perceptions of care quality. Significant EHR usability challenges and documentation burden have persisted as being reasons for why providers are frustrated with using their systems [52,53]. However, patients’ unmet expectations may increasingly become a consequence to providers’ obvious frustration and disengagement with the EHR. Primary care settings need implementable strategies to convey to patients the ways that technology is being leveraged to support their care, for example, by training and evaluating providers and staff on participative communication strategies that use screen sharing or integration of EHR task completion with patient engagement [31,54]. This may be especially necessary for providers caring for those with mistrust of the health care system and/or those with limited English proficiency or other communication or health literacy barriers, who may benefit from engaging with technology but may be wary of providers’ capacity to deliver high value care through the EHR. Policy efforts are also needed to encourage vendors to improve usability and development of features that meaningfully support the most cognitively challenging and time-intensive tasks in primary care [55]. Ongoing federal initiatives that focus on enabling use of third-party applications for self-management and patient-generated health data (ie, tools untethered to the EHR) need to also be accompanied by guidelines and support for integrating these tools in to practice.

### Future Work

As older adults’ options for technology-assisted health and health care continue to grow, shared expectations are increasingly important for how use of these tools will be supported and matched by adept technology use among providers. Future research should emphasize the salient contextual factors that influence technology use and technology perceptions particularly relevant to older adults’ health care utilization. For example, older adults are likely to receive support from family or informal caregivers, and caregivers’ perception of provider quality may influence their older relatives’ perception of quality. Future research should consider how the informal caregivers’ role could be best accommodated and facilitated in the presence of EHR-supported care delivery [56,57]. Simultaneously, providers and organizations should be considerate of the potential for additional burdens on caregivers to foster their older relative’s engagement with technology. Organizational changes required to meet these identified needs are not insignificant, but can build the essential capacity for patient-centered, value-based care.

### Limitations

This study has two key limitations. First, this survey is a cross-sectional analysis and we cannot make any causal claims about the impact of patient or provider use of technology on older adults’ assessment of care quality. Our research approach is descriptive and not designed to address the endogeneity of portal use with respect to health needs and the nature of recent health encounters. Second, the study uses a convenience sample and diverges on some key characteristics (ie, age distribution, educational achievement, rurality) relative to nationwide statistics. The sample also comprised mostly community-dwelling older adults and was primarily accessed online, excluding those older adults living in nursing home facilities, those with severe illnesses, and those with significant technology barriers. Our findings are thus not broadly generalizable, particularly for more clinically and socially complex older adult populations, but do provide important insights for the growing number of older adults who are interested and capable of participating in technology-supported health care.

### Conclusions

Despite significant barriers for many (ie, connectivity and individual comfort with technology), certain populations of older adults are using patient portals and updating their expectations about technology-enabled health care delivery at growing rates. This 2019 statewide survey is evidence for widespread but highly variable portal use. Older adults who are moderate portal users are the least satisfied with their care and the most sensitive to perceptions of how well their provider uses the EHR to support care. This offers preliminary evidence to inform an understanding of the link between patient technology use and perceptions of care quality, moderated by expectancy confirmation. Patients’ satisfaction with care at different chosen levels of portal use depends on whether providers offer an experience that aligns with their expectations. Encouraging older adults in more nascent use of patient portals may negatively affect perceptions of care quality if providers are not demonstrating adeptness with their own technology use during in-person visits and for asynchronous interaction. Organizationally, clinics need to consider changes to technology-enabled care—such as better access for, and integration of, caregivers—that are sensitive to, and accommodating of, the evolving needs and expectations of older adults. Ultimately, regulatory and payer policy changes are necessary to address the root causes of provider’s frustration and disengagement with technology-supported care practices, especially poor EHR usability and lack of support for integration and efficient use of portals or other remote technologies.

## Appendix

Multimedia Appendix 1 Survey questions for developing the quality composite measure.

Multimedia Appendix 2 Exploratory factor analysis.

Multimedia Appendix 3 Characteristics associated with portal use.

Multimedia Appendix 4 Variation in portal use, by number of medications taken and by self-reported health status.

## Abbreviations

CAHPS: Consumer Assessment of Healthcare Providers and Systems
EHR: electronic health record
