# Supplementary material for: Patient Portal Use, Perceptions of Electronic Health Record Value, and Self-Rated Primary Care Quality Among Older Adults: Cross-sectional Survey
Source: J Med Internet Res. 2021 May 10;23(5):e22549. doi: 10.2196/22549 (PMC8145092; doi:10.2196/22549)
Supplement: Multimedia Appendix 1 [file jmir_v23i5e22549_app1.docx]

Appendix Exhibit 1: Survey Questions for Developing Quality Composite Measures (Cronbach’s alpha= 0.94)

| **Survey Question** | **Source** |
| --- | --- |
| 1. My personal doctor shows respect for what I have to say (Respect) | Questions pulled from CAHPS Survey  <https://www.ahrq.gov/cahps/surveys-guidance/cg/about/survey-measures.html> |
| 2. My personal doctor listens carefully to me (Listens) |  |
| 3. My personal doctor explains things in a way that I can understand (Explains) |  |
| 4. My personal doctor spends enough time with me (Time) |  |
| 5. My personal doctor is trained in care for older adults (Trained) | Derived from focus group analysis to reflect age context |
| 6. My personal doctor has the skills to provide good care to someone my age (AgeSkills) |  |
| 7. My personal doctor understands that older adults have unique concerns and needs (AgeNeeds) |  |
| 8. My personal doctor trusts me (Trusts) |  |
| 9. My personal doctor is on the same page as any other doctors I may see (SamePage) | Derived from focus group analysis to reflect care coordination |
| 10. My personal doctor knows what information is important to tell my other doctors (SharesInfo) |  |
| 11 My personal doctor gives attention to both my physical and mental health (MentalPhysical) | Derived from focus group analysis to reflect patient-centeredness |
| 12. My personal doctor is responsive to my concerns (Responsive) |  |
| 13. My personal doctor has been able to correctly diagnose my conditions (Diagnose) |  |
