# Supplementary material for: Patient Portal Use, Perceptions of Electronic Health Record Value, and Self-Rated Primary Care Quality Among Older Adults: Cross-sectional Survey
Source: J Med Internet Res. 2021 May 10;23(5):e22549. doi: 10.2196/22549 (PMC8145092; doi:10.2196/22549)
Supplement: Multimedia Appendix 2 [file jmir_v23i5e22549_app2.docx]

Appendix Exhibit 2. Exploratory Factor Analysis

factor $QualityQuestions, ml factors(1)

(obs=152)

Iteration 0: log likelihood = -141.63624

Iteration 1: log likelihood = -124.04601

Iteration 2: log likelihood = -123.89803

Iteration 3: log likelihood = -123.88194

Iteration 4: log likelihood = -123.88018

Iteration 5: log likelihood = -123.87999

Iteration 6: log likelihood = -123.87997

Iteration 7: log likelihood = -123.87997

Factor analysis/correlation Number of obs = 152

Method: maximum likelihood Retained factors = 1

Rotation: (unrotated) Number of params = 13

Schwarz's BIC = 313.07

Log likelihood = -123.88 (Akaike's) AIC = 273.76

--------------------------------------------------------------------------

Factor | Eigenvalue Difference Proportion Cumulative

-------------+------------------------------------------------------------

Factor1 | 7.64072 . 1.0000 1.0000

--------------------------------------------------------------------------

LR test: independent vs. saturated: chi2(78) = 1513.15 Prob>chi2 = 0.0000

LR test: 1 factor vs. saturated: chi2(65) = 238.25 Prob>chi2 = 0.0000

Factor loadings (pattern matrix) and unique variances

---------------------------------------

Variable | Factor1 | Uniqueness

-------------+----------+--------------

Trained | 0.7802 | 0.3912

AgeSkills | 0.7004 | 0.5094

AgeNeeds | 0.8004 | 0.3593

SharesInfo | 0.7999 | 0.3601

MentalPhysical | 0.6847 | 0.5312

SamePage | 0.8396 | 0.2951

Diagnose | 0.8082 | 0.3467

Respect | 0.7457 | 0.4440

Listens | 0.7950 | 0.3679

Explains | 0.7390 | 0.4538

Responsive | 0.7756 | 0.3984

Time | 0.7615 | 0.4201

Trust | 0.7198 | 0.4819

---------------------------------------
