# Supplementary material for: Patient Portal Use, Perceptions of Electronic Health Record Value, and Self-Rated Primary Care Quality Among Older Adults: Cross-sectional Survey
Source: J Med Internet Res. 2021 May 10;23(5):e22549. doi: 10.2196/22549 (PMC8145092; doi:10.2196/22549)
Supplement: Multimedia Appendix 3 [file jmir_v23i5e22549_app3.docx]

Appendix Exhibit 3. Characteristics Associated with Portal Use

| Portal Use | N | None | 1-3 Uses | 4-7 Uses | P-value |
| --- | --- | --- | --- | --- | --- |
| Average age | 158 | 74.8 | 70.02 | 70.34 | **<0.001** |
| Male | 161 | 11 (20.8%) | 13 (24.5%) | 29 (54.7%) |  |
| Female |  | 24 (22.2%) | 36 (33.3%) | 48 (44.4%) |  |
| Does not have caregiver | 157 | 21 (19.1%) | 35 (31.8%) | 54 (49.1%) | 0.283 |
| Has caregiver |  | 14 (29.8%) | 11 (23.4%) | 22 (46.8%) |  |
| No PCP visit in last 6 months | 161 | 4 (16.0%) | 8 (32.0%) | 13 (52.0%) | 0.708 |
| PCP Visit in last 6 months |  | 32 (23.5%) | 40 (29.4%) | 64 (47.1%) |  |
| 1-3 total doctors seen in past 6 months | 157 | 26 (29.6%) | 28 (31.8%) | 34 (38.6%) | **0.019** |
| 4+ total doctors seen in past 6 months |  | 9 (13.0%) | 20 (29.0%) | 40 (58.0%) |  |
| Self Reported Health Fair/Worse | 152 | 10 (35.7%) | 4 (14.3%) | 14 (50.0%) | 0.06 |
| Self Reported Health Good |  | 8 (22.9%) | 14 (40.0%) | 13 (37.1%) |  |
| Self Reported Health Very Good/Excellent |  | 14 (15.7%) | 27 (30.3%) | 48 (53.9%) |  |
| Has seen PCP 10 or more years | 160 | 11 (23.4%) | 15 (31.9%) | 21 (44.7%) | 0.84 |
| Has seen PCP 5 - 10 years |  | 8 (18.6%) | 11 (25.6%) | 24 (55.8%) |  |
| Has seen PCP less than 5 years |  | 16 (22.9%) | 22 (31.4%) | 32 (45.7%) |  |
| No technology concerns | 164 | 25 (18.3%) | 43 (31.4%) | 69 (50.3%) | **0.01** |
| Has technology concerns |  | 12 (44.4%) | 7 (25.9%) | 8 (29.6%) |  |
| Have Money Sometimes/Never | 160 | 22 (40.7%) | 19 (35.2%) | 13 (24.1%) | **<0.001** |
| Have Money Always/Often |  | 13 (12.3%) | 29 (27.4%) | 64 (60.4%) |  |
| Taking 0 prescription medications | 161 | 3 (9.4%) | 12 (37.5%) | 17 (53.1%) | 0.118 |
| Taking 1-3 prescription medications |  | 17 (23.0%) | 18 (24.3%) | 39 (52.7%) |  |
| Taking 4+ prescription medications |  | 16 (29.1%) | 19 (34.6%) | 20 (36.4%) |  |
