# Supplementary material for: Patient Portal Use, Perceptions of Electronic Health Record Value, and Self-Rated Primary Care Quality Among Older Adults: Cross-sectional Survey
Source: J Med Internet Res. 2021 May 10;23(5):e22549. doi: 10.2196/22549 (PMC8145092; doi:10.2196/22549)
Supplement: Multimedia Appendix 4 [file jmir_v23i5e22549_app4.docx]

Appendix Exhibit 4a. Variation in Portal Use, by Number of Medications Taken


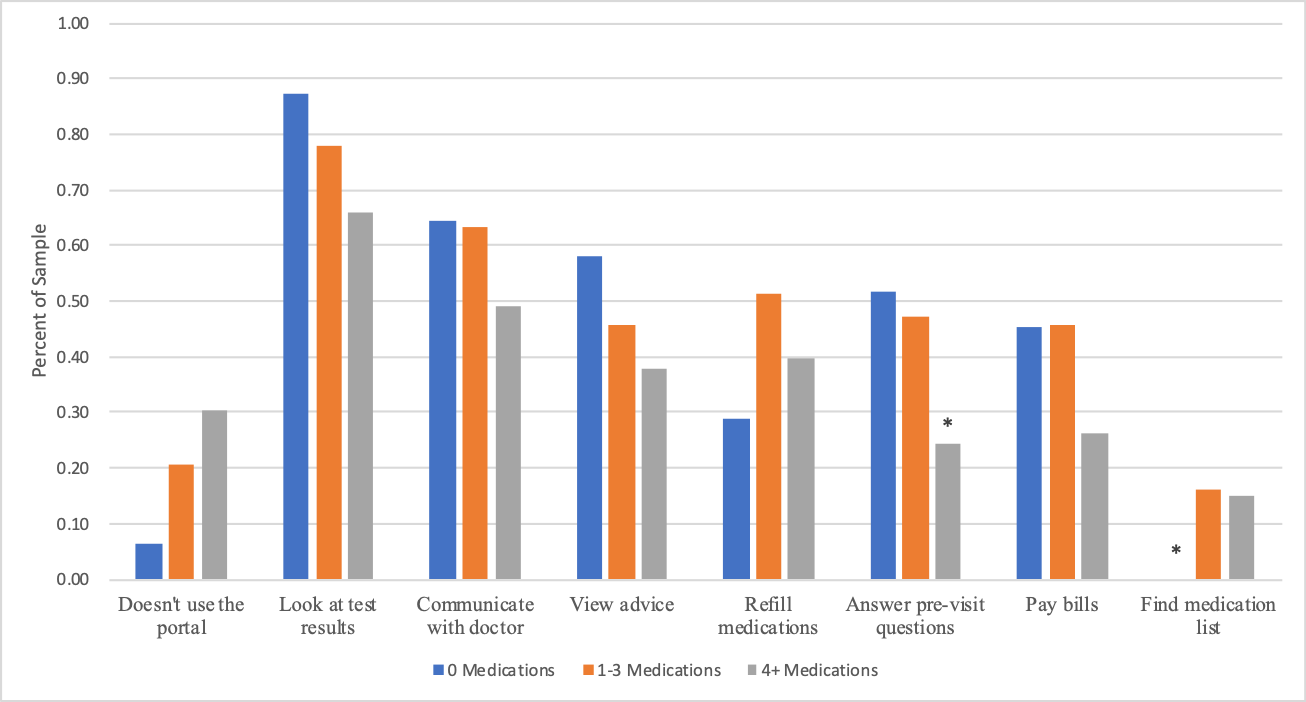


* p<0.05

Appendix Exhibit 4b. Variation in Portal Use, by Self-Reported Health Status
